# 404. Genomic surveillance reveals reduced Staphylococcus aureus transmission in semi-private versus multi-bed hospital rooms

**DOI:** 10.1093/ofid/ofaf695.142

**Published:** 2026-01-11

**Authors:** Courtney Takats, Sarah E Hochman, Alejandro Pironti, Bo Shopsin, Gregory Putzel, Magdalena M Podkowik, Alice Tillman, Julia Shenderovich, Natalia M Arguelles, Anusha Srivastava, Michael Phillips

**Affiliations:** NYU Langone Health, New York, New York; NYU Langone Health, New York, New York; NYU Grossman School of Medicine, New York, New York; NYU Langone Health, New York, New York; NYU Langone, New York, New York; New York University, New York, New York; NYU Langone, New York, New York; NYU Langone Health, New York, New York; Washington University School of Medicine, St. Louis, Missouri; Case Western Reserve University School of Medicine, Cleveland, Ohio; New York University, New York, New York

## Abstract

**Background:**

Although single-patient rooms are recommended for hospital design to reduce healthcare-associated infections, construction is costly, and supporting evidence remains limited. Most studies rely on pre/post time series analyses that cannot account for concurrent infection control and antimicrobial stewardship initiatives. To address this gap, we used comprehensive, hospital-wide whole-genome sequencing (WGS) surveillance to compare Staphylococcus aureus transmission rates in single- versus multi-bed rooms during routine clinical operations.

**Methods:**

Across two hospitals in an urban health system, ∼3,000 patients per month underwent active S. aureus surveillance using admission nasal swabs. Surveillance and clinical (blood, sputum, wound) isolates collected between October 2022 - December 2023 underwent WGS. Closely related isolates (< 20 single nucleotide polymorphisms) that were epidemiologically linked based on timestamped patient location data were classified as high-probability transmissions. We compared S. aureus transmission rates among single, two-bed, and four-bed rooms, all of which underwent identical cleaning and sporicidal disinfection protocols.

**Results:**

Over 14-months, >5,000 isolates from 4,000 patients underwent WGS, 85% of which were from surveillance cultures. Of admissions, 21% were to single rooms, 59% to two-bedded rooms, and 20% to four-bedded rooms. Transmission risk was lowest in single rooms (0.5 transmissions per 1000 admissions), increased in two-bed rooms (1.2 per 1000 admissions), and was nearly ninefold higher in four-bed rooms compared to single rooms (4.4 transmissions per 1000 admissions).

**Conclusion:**

In this large WGS-based surveillance study, single and semi-private (two-bed) rooms were associated with substantially lower S. aureus transmission rates compared to four-bed rooms. These findings indicate that room occupancy is a modifiable factor in transmission risk and that standard infection control practices may be insufficient; therefore, innovative, continuous disinfection strategies are warranted. Ongoing work is adjusting for potential differences in patient populations across room types to refine estimates of the impact of room occupancy on transmission.

**Disclosures:**

All Authors: No reported disclosures

